# Intrusion of Fukushima-derived radiocaesium into subsurface water due to formation of mode waters in the North Pacific

**DOI:** 10.1038/srep22010

**Published:** 2016-02-26

**Authors:** Hideki Kaeriyama, Yugo Shimizu, Takashi Setou, Yuichiro Kumamoto, Makoto Okazaki, Daisuke Ambe, Tsuneo Ono

**Affiliations:** 1 Research Center for Fisheries Oceanography and Marine Ecosystem, National Research Institute of Fisheries Sciences, Fisheries Research Agency, 2-12-4 Fukuura, Kanazawa, Yokohama, Kanagawa 236-8648, Japan; 2 Research and Development Center for Global Change, Japan Agency for Marine-Earth Science and Technology, 2-15 Natsuhima-cho, Yokosuka, Kanagawa 237-0061, Japan

## Abstract

The Fukushima Dai-ichi Nuclear Power Plant accident in March 2011 released radiocaesium (^137^Cs and ^134^Cs) into the North Pacific Ocean. Meridional transects of the vertical distribution of radiocaesium in seawater were measured along 147 °E and 155 °E in October–November 2012, 19 months after the accident. These measurements revealed subsurface peaks in radiocaesium concentrations at locations corresponding to two mode waters, Subtropical Mode Water and Central Mode Water. Mode water is a layer of almost vertically homogeneous water found over a large geographical area. Here we show that repeated formation of mode water during the two winter seasons after the Fukushima accident and subsequent outcropping into surface water transported radiocaesium downward and southward to subtropical regions of the North Pacific. The total amount of Fukushima-derived ^134^Cs within Subtropical Mode Water, decay-corrected to April 2011, was estimated to be 4.2 ± 1.1 PBq in October–November 2012. This amount of ^134^Cs corresponds to 22–28% of the total amount of ^134^Cs released to the Pacific Ocean.

The ocean is a large reservoir, not only for water, heat, and marine organisms, but also for anthropogenic substances, including radionuclides. After the Tohoku earthquake of 11 March 2011 (moment magnitude 9.0) and the subsequent tsunami, a loss of electric power at the Fukushima Dai-ichi Nuclear Power Plant (hereafter FNPP) resulted in overheated reactors and hydrogen explosions. Radioactive materials were then released into the ocean through both atmospheric deposition and leakage of heavily contaminated coolant water^1^,^2^. Because the half-lives of the radiocaesium isotopes ^134^Cs and ^137^Cs are 2.07 years and 30.07 years, evaluation of this radiocaesium in the marine environment is important for addressing risks to both public health through consumption of fisheries products and to marine ecosystems^3^,^4^. Total deposition of FNPP-derived radiocaesium had been estimated based on the observational data and simulation models with a considerable variation^5^,^6^,^7^,^8^,^9^,^10^,^11^,^12^,^13^. At the date of this writing, the latest estimate of the total input of FNPP-derived radiocaesium into the Pacific Ocean is 3–4 PBq via direct discharge and 12–15 PBq via atmospheric deposition^14^.

The dispersion patterns of FNPP-derived radiocaesium have been well documented in surface seawater^15^,^16^,^17^,^18^,^19^,^20^,^21^, but understanding the distribution of radiocaesium throughout the water column is essential for assessing its impact on marine ecosystems. The coastal location of the FNPP (37°25′N, 141°2′E) is near the Kuroshio current and its extension, which together constitute the western and northern part of the North Pacific subtropical gyre. Many researchers have argued that radiocaesium from the FNPP was dispersed eastward, north of the Kuroshio Extension (KE)^17^,^18^,^19^,^20^,^21^. In fact, FNPP-derived radiocaesium was detected in June 2013 at a concentration of about 0.7 Bq m^−3^ off the coast of British Columbia^21^. This concentration is, nevertheless, 1–2 orders of magnitude smaller than concentrations observed in the western North Pacific. The KE was thought to prevent southward dispersion of radiocaesium in surface water. The southward dispersion of the FNPP-derived radiocaesium in surface seawater was not reported during March 2011 and August 2012, probably because the oceanic front associated with a strong KE transported the FNPP-derived radiocaesium far eastward before it was dispersed southward^15^,^17^,^18^.

However, there was atmospheric deposition of radiocaesium onto the area south of the KE^17^,^22^,^23^. Some papers have reported dispersion of FNPP-derived radiocaesium in subsurface waters south of the KE, but the data are limited spatially and temporally^24^,^25^,^26^,^27^,^28^,^29^,^30^. Our previous study documented southward transport of FNPP-derived radiocaesium as far as 18 °N, 135 °E in subsurface waters by September 2012, 18 months after the FNPP accident^24^. Before the FNPP-accident, subsurface peaks of bomb-derived ^137^Cs were reported in Subtropical Mode Water (STMW) and Central Mode Water (CMW) at 20 °N along 165 °E^31^. Mode water is a layer of almost vertically homogeneous water found over a large geographical area. Mode water is formed as a result of deep winter mixing and is capped by the seasonal thermocline in spring^32^. The STMW is formed along the Kuroshio and its extension during winter to spring and then it spreads southward along the Kuroshio recirculation. The newly formed STMW can reach southern part of 137 °E section half a year after the formation^33^. The subsurface peaks of FNPP-derived radiocaesium were also located in the STMW and CMW^24^,^25^,^26^,^27^,^28^,^29^,^30^. Preliminary estimates of the total amount of radiocaesium trapped in the STMW are 6 PBq during March and April 2011 based on observations from December 2011 to February 2012^27^, and an additional 5 PBq entered prior to April 2012 during the winter of 2011–2012 based on observations during September 2012^24^. These estimates are based on limited data that were obtained from a single meridional transect along 149 °E^27^ or in the western part of the STMW (135 °E and 138 °E)^24^, and spatial heterogeneity of radiocaesium was not considered. The estimated values are therefore associated with large uncertainties.

Here we report vertical profiles of radiocaesium at 37 stations along two meridional transects at 147 °E and 155 °E during October–November 2012. The data at nine stations between 30°30′N and 36°30′N along 147 °E have been reported previously^24^,^34^. Using previously published data obtained in the area of 12–30 °N, 135–139 °E in September-October 2012^24^, 8–40 °N along 165 °E in June 2012^30^, where correspondence to downstream region of STMW, and the data from this study, which included the formation area of STMW, we estimated the total amount of FNPP-derived radiocaesium in the STMW 19 months after the FNPP accident, after two periods of winter mixing. Because the subsurface vertical minimum layer of potential vorticity (PV) less than 2.0 × 10^−10^ m^–1^ s^–1^ represents the distribution of STMW rather well^32^,^33^, the correlation between radiocaesium concentrations and PV allowed us to precisely estimate the total amount of FNPP-derived radiocaesium in the STMW.

## Results

### Vertical profiles of ^134^Cs and ^137^Cs activity

**Figure 1:**
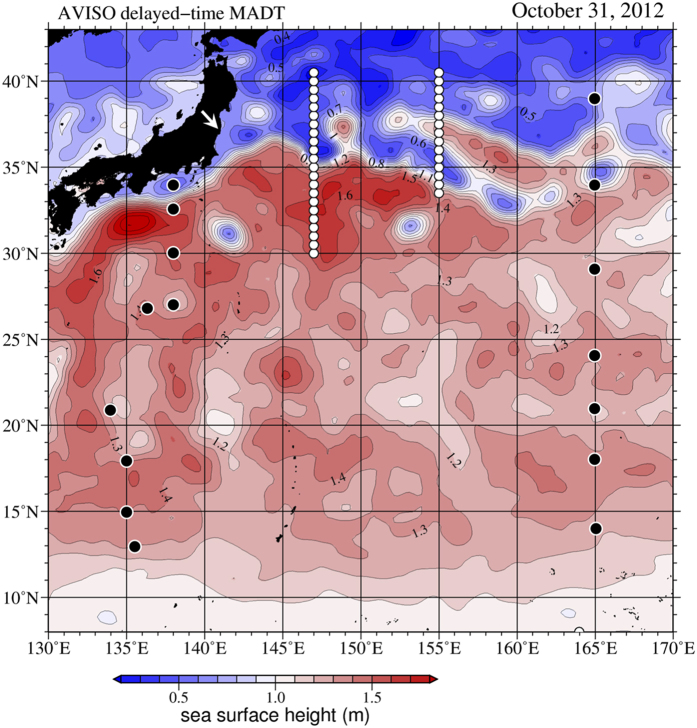
Sea surface height (SSH) of the Pacific Ocean near Japan. Sampling locations for radiocaesium measurements during October and November 2012 (white circles along the 147 °E and 155 °E transects) are shown. Black circles denote stations of previously reported studies, 12–30 °N, 135–139 °E in September-October 2012^24^, and 8–40 °N along 165 °E in June 2012^30^. The white arrow shows the location of the Fukushima Dai-ichi Nuclear Power Plant. The SSH data is based on one-week average gridded data (1/4° × 1/4°) for 31 October 2012; they were produced by the Segment Sol Multimissions d’Altimétrie d’Orbotographie et de Localisation Précise/Data Unification and Altimeter Combination System and distributed by the Archiving, Validation and Interpretation of Satellites Oceanographic Data with support from the Centre National d’Etudes Spatiales (http://www.aviso.altimetry.fr/duacs/). The map was made by using the General Mapping Tools version 4.5.11 (http://gmt.soest.hawaii.edu).

Our study area in the western North Pacific extended from subarctic to subtropical waters. The image of sea surface height (SSH) on 31 October 2012 produced from satellite data revealed a steep SSH gradient that implied a strong KE was flowing eastward at around 35 °N at the 147 °E meridian and around 33°30′N at the 155 °E meridian (Fig. 1). We define areas north and south of the KE front as transitional and subtropical regions, respectively. The transitional region lies between the subarctic Oyashio current and the KE^27^. The SSH distribution also suggested that a strong northward meander of the KE was present in the transition region between 36 °N and 38 °N at the 155 °E meridian (see also Fig. S1).

**Figure 2:**
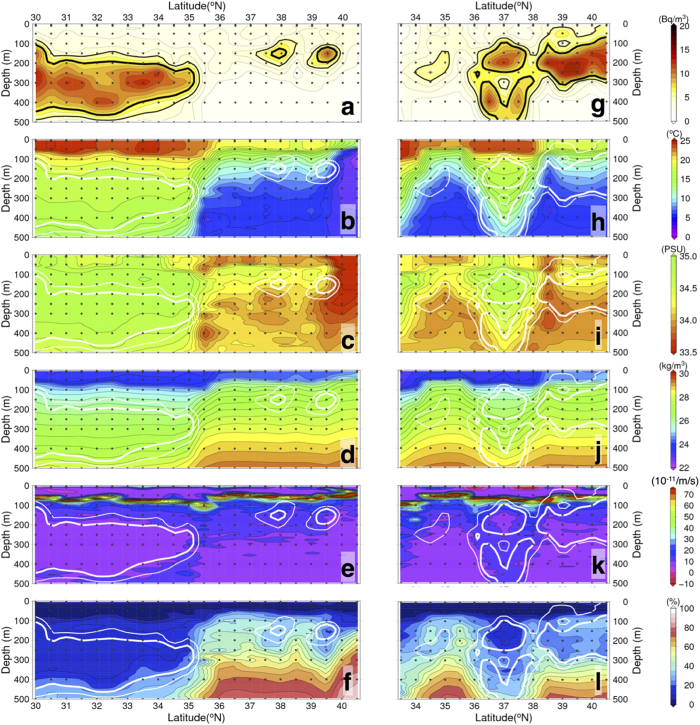
Latitudinal depth profiles. (**a**–**f**) Profiles along 147 °E and (**g**–**l**) profiles along 155 °E showing (**a**,**g**) ^137^Cs activity (Bq m^−3^), (**b**,**h**) potential temperature (°C), (**c**,**i**) practical salinity, (**d**,**j**) potential density (kg m^−3^), (**e**,**k**) potential vorticity (×10^−11^ m^−1^ s^−1^), and (**f**,**l**) apparent oxygen utilization (%) during October and November 2012. Black dots show where water samples for radiocaesium analysis were collected. Bold lines (black in (**a**,**g**) and white in other panels) indicate isolines of 5 Bq m^−3^ and 7 Bq m^−3^ of ^137^Cs activity, respectively. Data on ^137^Cs activity, potential temperature, practical salinity, potential density, potential vorticity and AOU in this figure are listed in Supplementary Table S1 together with ^134^Cs activity data. The maps were made by using the General Mapping Tools version 4.5.11 (http://gmt.soest.hawaii.edu).

**Figure 3:**
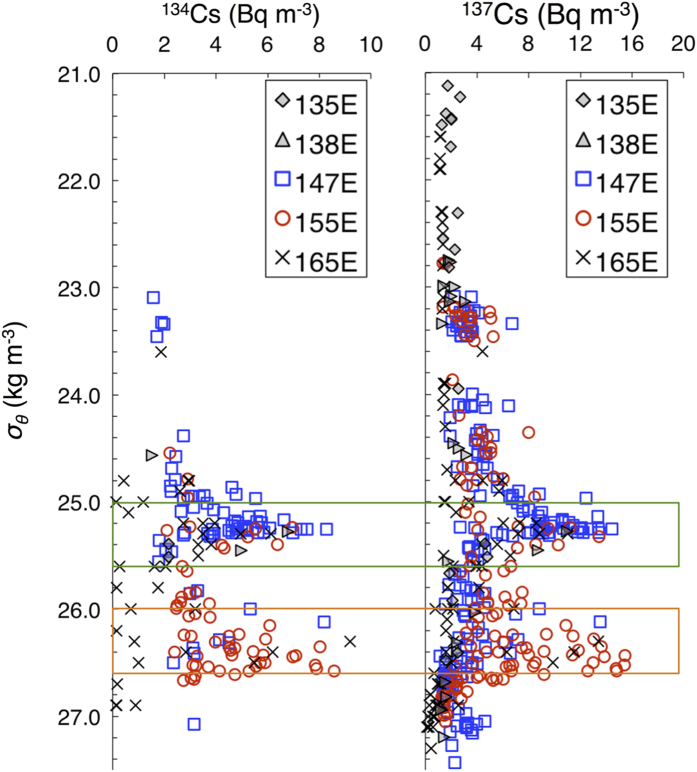
Profiles of ^134^Cs and ^137^Cs activity in relation to potential density. Blue squares and red circles indicate activities obtained along 147 °E and 155 °E, respectively, during October–November 2012. Grey diamonds and triangles indicate activities obtained along approximately 135 °E from 12 °N to 25 °N in September 2012 and along 135 °E from 27 °N to 30 °N in October 2012, respectively^24^. Crosses indicate activities obtained along 165 °E from 8 °N to 40 °N in June 2012^30^. Green and orange boxes indicate ranges of potential density of STMW (25.0–25.6 *σ*_*θ*_) and CMW (26.0–26.6 *σ*_*θ*_), respectively^35^.

The ^134^Cs/^137^Cs ratio, decay-corrected to 6 April 2011, the date of the largest leakage from the FNPP^2^, was not significantly different from 1.0 (0.9 ± 0.2), and the patterns of vertical profiles of ^134^Cs and ^137^Cs were almost the same at each station (Fig. S2), the indication being that most of the radiocaesium was derived from the FNPP^2^. At most of the sampling stations, the concentration of ^137^Cs was higher in subsurface water than at the surface (Fig. 2a,g). Peaks of ^137^Cs were observed at depths of 300–400 m between 30 °N and 40 °N along the 147 °E transect, and at depths of about 200 m between 38°30′N and 40°30′N and in patches around the KE front (36°30′N–38 °N) along the 155 °E transect (Fig. 2a,g; see Supplemental Table S1 for ^134^Cs data). Subsurface peaks of ^137^Cs were observed in water masses with low potential vorticity (lower than 2.0 × 10^−10^ m^−1^ s^−1^: Fig. 2e,k) and potential density ranges of 25.0–25.6 *σ*_*θ*_ and 26.0–26.6 *σ*_*θ*_ (Fig. 3). These characteristics of the subsurface ^137^Cs peaks, as well as other water properties such as potential temperature and salinity (Fig. 2), suggest that the FNPP-derived radiocaesium was associated with Subtropical Mode Water (STMW; 25.0–25.6 *σ*_*θ*_) and Central Mode Water (CMW; 26.0–26.6 *σ*_*θ*_)^35^.

Along the 147 °E transect, FNPP-derived radiocaesium was distributed mostly in STMW in the subtropical region, whereas along the 155 °E transect the main radiocaesium peaks were within the density range of CMW in the transitional region mainly due to the lack of observations in the formation area of STMW where the southern recirculation of KE^32^,^33^. The 155 °E transect revealed a complex distribution of water masses in the transitional area, especially just north of the KE. Two subsurface peaks of ^137^Cs were observed at 37 °N at depths of 200 m and 500 m. The upper peak was within STMW. The peak at a depth of 500 m had a potential density of 26.4 *σ*_*θ*_, which is in the range of CMW (Fig. 3). At adjacent stations along the transect, we observed only one subsurface peak of ^137^Cs, which was within CMW. The CMW with FNPP-derived radiocaesium may have been subducted deep water below the STMW layer at 37 °N since the CMW cross-frontal subduction^36^,^37^, possibly via strong meandering of the KE, which would become an anticyclonic eddy (Fig. S1). Unlike adjacent stations, at 37 °N we therefore found double peaks of ^137^Cs corresponding to STMW and CMW. North of 38 °N along the 155 °E transect, the FNPP-derived radiocaesium core was within CMW. These findings are consistent with those of previous studies^25^,^26^,^27^, which have reported the detection of ^134^Cs in STMW around 149 °E in the winter of 2012, in STMW at 137 °E during April of 2012 and March of 2013^24^, and in CMW at 165 °E in June of 2012^30^. Concentrations of ^137^Cs in the subsurface peaks in this and previous studies have also been roughly comparable, ranging between 5 and 15 Bq m^−3^ (Fig. 3)^24^,^25^,^26^,^27^,^28^,^30^.

### Spatial variation of the water column inventory of ^137^Cs

**Figure 4:**
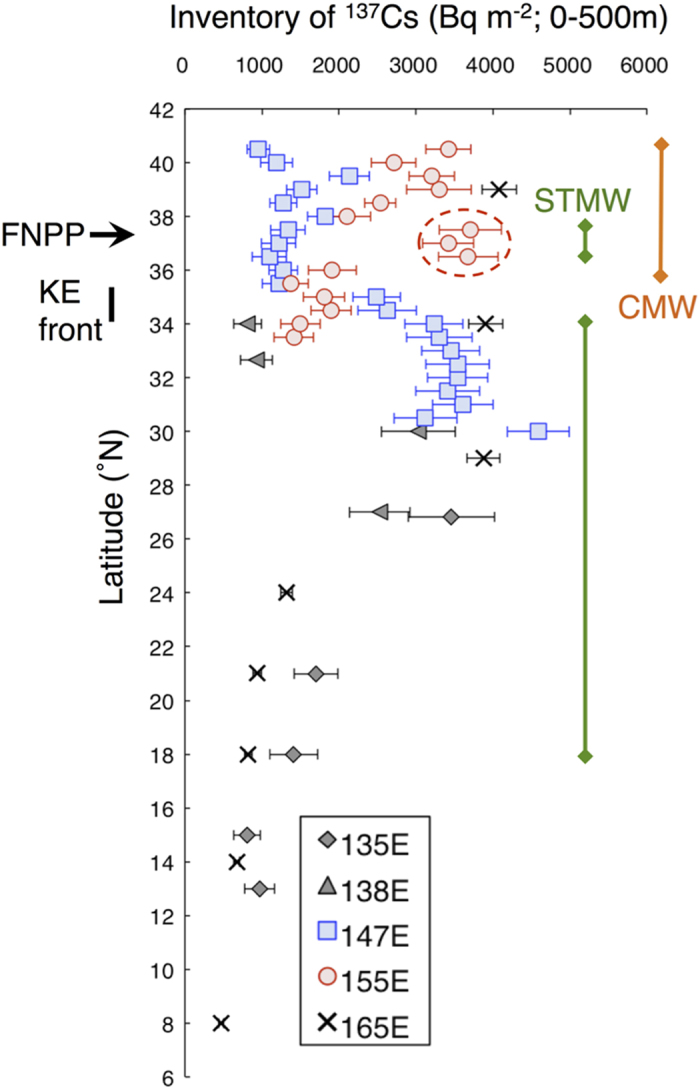
Vertically integrated inventories of ^137^Cs. Inventories are integrated between the surface and 500 m depth in the western North Pacific in the summer and autumn of 2012. Symbols are the same as in Fig. 3. Error bars indicate uncertainties (standard deviations). The positions of the FNPP and the KE front in October–November 2012 are indicated on the left side. Green and orange bars indicate approximate positions of STMW and CMW in October–November 2012. The red dashed circle indicates the area of strong meandering by the KE around 155 °E.

The water column inventory of ^137^Cs between the surface and a depth of 500 m ranged from 960 ± 150 to 4590 ± 400 Bq m^−2^ (Fig. 4). The highest and lowest inventory values were observed at the southernmost (30 °N) and northernmost stations (40°30′N), respectively, along the 147 °E transect. The inventories of ^137^Cs at most sampling stations in this study were greater than those before the FNPP accident in the North Pacific (~1000 Bq m^−2^)^28^,^38^,^39^. Areas of mode waters had ^137^Cs inventories exceeding 2000 Bq m^−2^. Examples include 30–35 °N along the 147 °E transect and 34°–40°30′N along the 155 °E transect. The KE appears to be the boundary between high and low ^137^Cs values, with high inventories south of the KE along the 147 °E transect and high inventories north of the KE along the 155 °E transect. Satellite images of SSH (Fig. 1) suggest that the KE was meandering strongly during the study period and had started meandering just before our field observations (Fig. S1). The area of high ^137^Cs inventories in STMW between 36°30′N and 37°30′N along the 155 °E transect originated in subtropical waters south of the KE, where radiocaesium was trapped. To the north of the KE, high inventories were also observed at stations where the subsurface peaks of ^134^Cs and ^137^Cs were associated with CMW, at 38 °N and 39°30′N along the 147 °E transect and at 38 °N–40°30′N along the 155 °E transect. Previous studies have documented a southward decrease in ^137^Cs inventories between 30°N and 8 °N (Fig. 4)^24^,^30^. In the western North Pacific, the peak area of Fukushima-derived radiocaesium was associated with areas of formation of mode water about 19 months after the FNPP accident, primarily in the subtropical region between 25 °N and 34 °N, where STMW formed, and secondarily in the transition area, where CMW formed (Fig. 4).

### Estimation of total amount of FNPP-derived radiocaecium in STMW during October–November 2012

**Figure 5:**
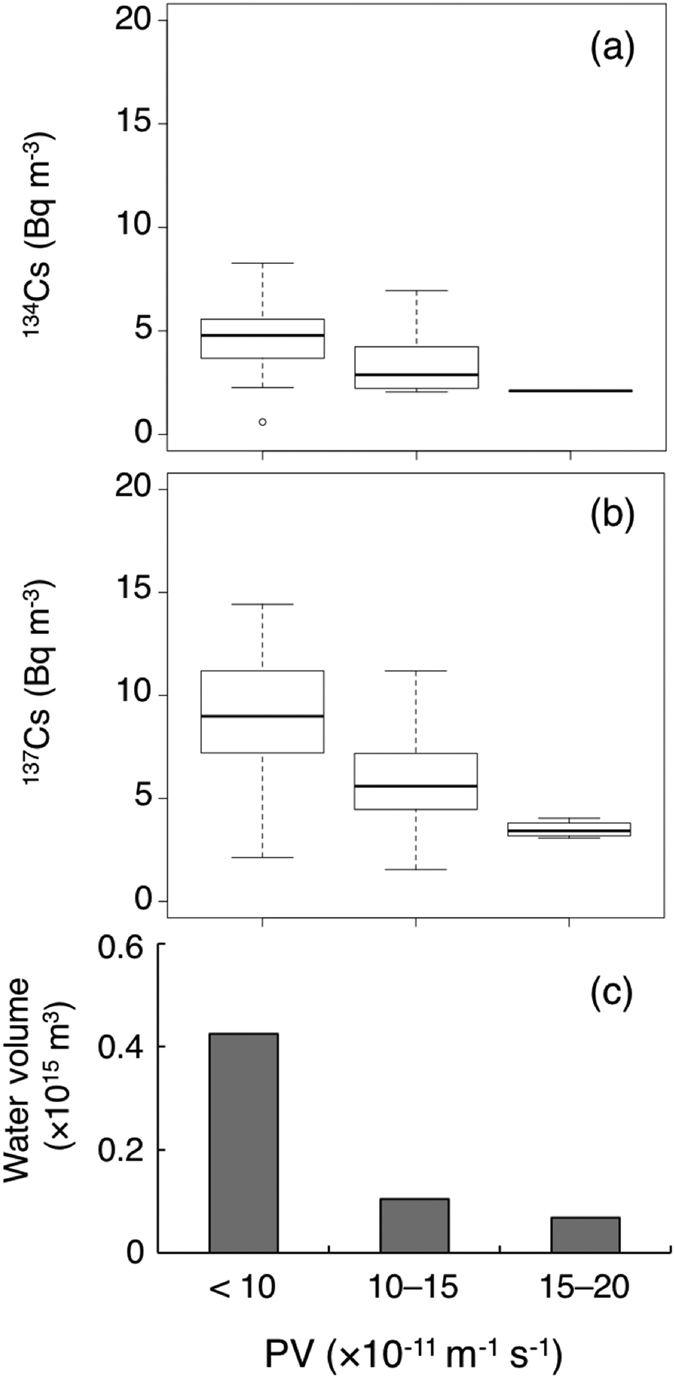
Correlation between radiocaesium and potential vorticity in STMW. Box plot of (**a**,**b**) ^134^Cs and ^137^Cs activities (Bq m^−3^) and (**c**) water volume (m^3^) of three potential vorticity classes (<1.0, 1.0–1.5 and 1.5–2.0 × 10^−10^ m^−1^ s^−1^). The radiocaesium activity data were obtained from those with the potential density range of 25.0–25.6 *σ*_*θ*_ in this study and data obtained during June and October 2012^24^,^30^. The water volume of each PV class was calculated based on the quality checked Argo data during October 2012, which were obtained from http://www.jamstec.go.jp/ARGO/argo_web/argo/index.html.

The total amount of radiocaesium in STMW in October–November of 2012 was estimated based on the average concentration of ^134^Cs and ^137^Cs (Bq m^−3^) and the volume of STMW water (m^3^) based on PV data lower than 2.0 × 10^−10^ m^−1^ s^−1^ calculated from ARGO profiling data. A negative correlation was observed between PV and ^137^Cs concentration in the whole region of STMW, not only from the formation region, but also from the southern region (Fig. S3). The STMW was divided into three PV classes, <1.0, 1.0–1.5 and 1.5–2.0 × 10^−10^ m^−1^ s^−1^, depending on the radiocaesium data, see details for Methods section (Fig. 5). The volume of water in each PV classes (<1.0, 1.0–1.5 and 1.5–2.0 × 10^−10^ m^−1^ s^−1^) was 0.42, 0.11 and 0.068 × 10^15^ m^3^, respectively. The total volume of STMW was 0.6 × 10^15^ m^3^, and the value is well consistent with estimation of Oka *et al.*^40^ The average concentrations of ^134^Cs within the STMW in the three PV classes were 4.7 ± 1.5, 3.5 ± 1.6 and 2.1 Bq m^−3^, and those of ^137^Cs were 9.2 ± 2.7, 6.1 ± 2.6 and 3.5 ± 0.4 Bq m^−3^, respectively. Only one data of ^134^Cs was available in the PV class of 1.5–2.0 × 10^−10^ m^−1^ s^−1^. The total amounts of ^134^Cs and ^137^Cs in STMW were calculated to be 2.5 ± 0.64 PBq and 4.8 ± 1.2 PBq, respectively. The amount of ^134^Cs in STMW in October–November 2012 was decay-corrected to April 2011. This calculation yielded an original ^134^Cs input of 4.2 ± 1.1 PBq, which accounted for 22–28% of the total release of ^134^Cs from the FNPP accident (15–19 PBq)^14^.

## Discussion

In contrast to the eastward dispersion and dilution of FNPP-derived radiocaesium in surface seawater, FNPP-derived radiocaesium concentrations in subsurface mode waters were higher than those in surface seawater 19 months after the FNPP accident. Our observations showed that a large amount of FNPP-derived radiocaesium existed in the subtropical region south of the KE 19 months after the FNPP accident. Subsurface peaks of FNPP-derived radiocaesium associated with STMW have also been reported during December 2011 and February 2012 on a transect along 149 °E^27^, in December 2011 at 20–30 °N on a transect along 135 °E^26^, between August 2011 and March 2013 far south of the Japanese islands around 138 °E^24^, and in June 2012 off the coast of Taiwan^29^. FNPP-derived radiocaesium has also been reported in CMW in the transitional region in June 2012^30^.

Both STMW and CMW form as a deep winter mixed layer during the winter, and thus the apparent oxygen utilization (AOU) is relatively small in these waters (Fig. 2f,i)^32^. Kumamoto *et al.*^25^ first reported a subsurface peak in FNPP-derived radiocaesium in the subtropical region at 149 °E in February 2012. They concluded that the subsurface peak of FNPP-derived radiocaesium that they observed in February 2012 was formed during winter-spring just after the FNPP accident (during March and April of 2011)^27^. On the basis of temporal changes in the subsurface peak of ^137^Cs, the detection of ^134^Cs, and the ^137^Cs inventory in the western part of the STMW area, Kaeriyama *et al.*^24^ have also reported an increase of FNPP-derived radiocaesium after the second winter-spring time interval (February and April of 2012). Other researchers have reported that the FNPP-derived radiocaesium plume in surface water was dispersed eastward in the area north of KE, where north of STMW formation region during the first year after the FNPP accident^15^,^17^,^18^,^30^. In fact, the compiled published data during August and December 2012 clearly reveal a plume of FNPP-derived ^134^Cs in surface water located in the center of the North Pacific (160 °E–170 °W)^41^. These findings suggest that the formation of the subsurface peak of Fukushima-derived radiocaesium associated with STMW occurred primarily during the two winter-spring time intervals following the accident (before April 2012). Because our observations were made two winter seasons after the FNPP accident, our dataset should be representative of the concentrations of FNPP-derived radiocaesium in STMW.

Preliminary estimates reveal that 6 PBq of FNPP-derived radiocaesium entered STMW during the first winter-spring time interval after the FNPP accident (March and April 2011)^27^, and an additional 5 PBq entered STMW during the second winter-spring time interval (until April 2012)^24^. These estimates are associated with large uncertainties, because they rely on limited data and do not consider the vertical profile of radiocaesium in STMW. Because our estimation of the total amount of FNPP-derived radiocaesium are based on data obtained from a broad area of STMW (135 °E–165 °E) and because spatial variations are taken into consideration based on the volume of STMW estimated from PV, our estimated value should be more accurate than values estimated in previous studies^24^. The present estimated total amount of radiocaesium in STMW probably represents the results of two winter-spring periods of deep mixing (March–April 2011 and February–April 2012), as well as one period of outcropping in surface water during the second winter-spring time interval (February–April 2012). These events were followed during the warm season by separation of subsurface water from surface water. These deep-mixing and outcrop processes resulted in the distribution of 2.5 and 4.8 PBq of FNPP-derived ^134^Cs and ^137^Cs in STMW 19 months after the FNPP accident.

The volume of STMW showed interannual as well as seasonal fluctuations^40^,^42^. Formation of STMW was 50% greater during the period of stable KE after 2010 than during the unstable KE period of 2006–2009^40^. Had the FNPP accident occurred during the unstable KE period, radiocaesium would have stayed in the western area by the eddy activities, resulting in its higher concentration in the shallower STMW layer. The remaining FNPP-derived radiocaesium would have been transported eastward in surface waters north of the KE and been diluted^17^,^18^,^20^,^21^.

Our estimate of how much Fukushima-derived radiocaesium entered the ocean interior is an underestimate, because we did not estimate the radiocaesium inventory in CMW, which would entrain Fukushima-derived radiocaesium to greater depths than STMW and outcrops at the surface in fewer locations^43^. The distribution of radiocaesium in CMW should be monitored in the future. Better understanding of the physical mechanisms behind the spatial and temporal distribution of CMW^36^,^37^,^44^,^45^ is important to understanding the fate of Fukushima-derived radiocaesium in the ocean interior. In other words, our findings should advance understanding of the physics of mode waters, one of the key features of oceanology and global climate change studies in terms of atmosphere-ocean interactions and heat flux.

## Methods

### Sample collection

Seawater samples were collected in 12 L Niskin-X bottles (General Oceanics, Inc.) at the stations shown in Fig. 1. Seawater samples were collected from 10 depths between 5 and 500 m during October and November 2012 by the *R/V Shoyo-maru*. Additional samples from depths of 750 and 1000 m were collected at three sampling stations around the KE axis at 34 °N, 35 °N, and 36 °N along the 147 °E meridian (Supplemental Table S1). Unfiltered seawater samples were transferred into a 20 L plastic bag and acidified to pH 1.6 by adding 40 mL of concentrated nitric acid. In the open ocean, differences in radiocaesium activity between filtered and unfiltered seawater were negligible^2^,^22^. Thus, we assume that radioactivity determined in this study is derived from dissolved radiocaesium in the seawater. Water properties including temperature (T), salinity (S), and dissolved oxygen (DO) data were also obtained every 1 dbar by a CTD unit (T, SBE3; S, SBE4; DO, SBE43; and depth, SBE9plus; Seabird Co., USA) at each sampling station. The salinity sensor was calibrated against bottled seawater, the salinity of which had been measured by a salinometer (Autosal model 8400B, Guildline Instruments). Potential vorticity (PV) is defined as


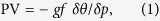


where *f* is the Coriolis parameter, *g* is the gravitational acceleration, *p* is pressure and *θ* is potential temperature. In equation (1), relative vorticity is neglected^44^,^46^. The apparent oxygen utilization (AOU), which is converted to the consumption ratio to the saturation at *in-situ* pressure, T, and S, is calculated using the DO sensor value.

### Analysis of ^134^Cs and ^137^Cs in seawater

The ^134^Cs and ^137^Cs in seawater samples were concentrated by adsorption onto ammonium phosphomolybdate (AMP) using a modified version of a method described elsewhere^18^,^24^. First, 0.52 g of CsCl was added to the sample as a carrier and then 8.0 g of AMP was added to the sample, which was then stirred for at least 1 h. After the AMP settled, the supernatant was decanted and the AMP/Cs compound was collected onto a glass fibre filter (GA-100, Advantec Co., Ltd.) by filtration and washed with nitric acid. The AMP/Cs compound was dried at 60–70°C for more than 48 h and then weighed. The average yield of the AMP/Cs compound was 95.6 ± 1.7 wt% (*n* = 375). The chemical yield was not determined, and 100% chemical yield was assumed (see details in the supplement file of Kaeriyama *et al.*)^24^. A high-purity coaxial germanium (HPGe) semiconductor detector with multichannel analyser (Seiko EG & G, Ortec, USA) measured the ^134^Cs and ^137^Cs activities in the AMP/Cs compounds. The detector had a resolution of 1.44 keV at a peak of 662 keV (^137^Cs), and relative efficiency was 33.0%. The energy-dependent efficiency calibration for the detector was conducted with five gamma-ray reference sources in a 100 mL plastic container identical to that used for sample measurement (Japan Radioisotope Association). The ^134^Cs and ^137^C activities were determined by analysing the photopeak area corresponding to 605 and 796 keV for ^134^Cs and 662 keV for ^137^Cs, obtained by counting for longer than 80,000 s at the laboratory of National Research Institute of Fisheries Sciences, Japan. Activities of ^134^Cs and ^137^C were corrected for decay since the sampling date. Coincidence summing effects of ^134^Cs were corrected with ^134^Cs standard solutions obtained from the Japan Radioisotope Association. Activity concentrations three times the standard deviation from counting statistics were defined as the detection limit concentrations. The detection limit of ^137^Cs with an 82,000 s count was nearly 1.3 Bq m^−3^, which was almost the same as was obtained in North Pacific surface water prior to the FNPP accident (1.0–2.5 Bq m^−3^)^38^,^39^. The water column inventory of ^137^Cs from 0 to 500 m depth (Bq m^−2^) was calculated from the gross vertical profile area using concentration data at each sampling depth. When ^137^Cs was not detected, we used the detection limit for calculations, and thus the inventories may be overestimated.

### Estimation of total amount of ^134^Cs and ^137^Cs in STMW

The total amount of radiocaesium in STMW in October–November of 2012 was estimated based on the average concentration of ^134^Cs and ^137^Cs (Bq m^−3^) and the volume of STMW water (m^3^). The radiocaesium data obtained in the area of 12–30 °N, 135–139 °E in September-October 2012^24^, 8–40 °N along 165 °E in June 2012^30^ and this study were used. The subsurface vertical minimum layer of PV less than 2.0 × 10^−10^ m^−1^ s^−1^ is a good representation of the distribution of STMW^32^. FNPP-derived radiocaesium within STMW was observed in the PV range of 0.5–1.8 × 10^−10^ m^−1^ s^−1^ (Fig. 2e,k) and a negative correlation was observed between PV and ^137^Cs (*r* = −0.63, *p* < 0.001, n = 75; Fig. S3). Figure S3 included data obtained not only from the formation region of STMW (30–38 °N along 145 °E and 155 °E), but also from the southern region of STMW along 135 °E and 165 °E, including the southernmost stations at 15 °N, 135 °E and 18 °N, 165 °E. Therefore, the significant negative correlation between PV and ^137^Cs is probably observed in the whole region of STMW.

According to Oka *et al.*^40^, the water volume of STMW was calculated based on the area of which PV less than 2.0 × 10^−10^ m^−1^ s^−1^ and the core potential temperature range of 16–19.5 °C using the quality checked Argo data during November 2012 in the interested area, which are vertically interpolated on to a 1 dbar from discrete set each profile. The STMW area was divided into the three PV class, <1.0, 1.0–1.5 and 1.5–2.0 × 10^−10^ m^−1^ s^−1^, respectively (Fig. 5). The average concentration of radiocaesium (Bq m^−3^) and water volume (m^3^) of each PV class were multiplied to obtain the total inventory of radiocaesium in each PV-class water mass. The total amount of FNPP-derived radiocaesium in STMW was equated to the total of the radiocaesium inventories of the three PV classes. The uncertainty of the estimated amount of radiocaesium was expressed based on the standard deviation obtained from the average concentration of radiocaesium in each PV class.

## Additional Information

**How to cite this article**: Kaeriyama, H. *et al.* Intrusion of Fukushima-derived radiocaesium into subsurface water due to formation of mode waters in the North Pacific. *Sci. Rep.* **6**, 22010; doi: 10.1038/srep22010 (2016).

## Supplementary Material

Supplementary Information

Supplementary Table S1
