# Supplementary material for: Intrusion of Fukushima-derived radiocaesium into subsurface water due to formation of mode waters in the North Pacific
Source: Sci Rep. 2016 Feb 26;6:22010. doi: 10.1038/srep22010 (PMC4768088; doi:10.1038/srep22010)
Supplement: Supplementary Information [file srep22010-s1.pdf]

Supplemental

Intrusion of Fukushima-derived radiocaesium into the subsurface water due to formation of mode waters in the North Pacific

Hideki Kaeriyama[1*], Yugo Shimizu[1], Takashi Setou[1], Yuichiro Kumamoto[2], Makoto Okazaki[1], Daisuke Ambe[1] and Tsuneo Ono[1]

[1]Research Center for Fisheries Oceanography and Marine Ecosystem, National Research Institute of Fisheries Sciences, Fisheries Research Agency, 2-12-4 Fukuura, Kanazawa, Yokohama, Kanagawa 236-8648, Japan

[2] Research and Development Center for Global Change, Japan Agency for Marine-Earth Science and Technology, 2-15 Natsuhima-cho, Yokosuka, Kanagawa 237-0061, Japan

* Corresponding author: Hideki Kaeriyama

Tel: +81-45-788-7654
Fax: +81-45-788-5001
E-mail: kaeriyama@affrc.go.jp

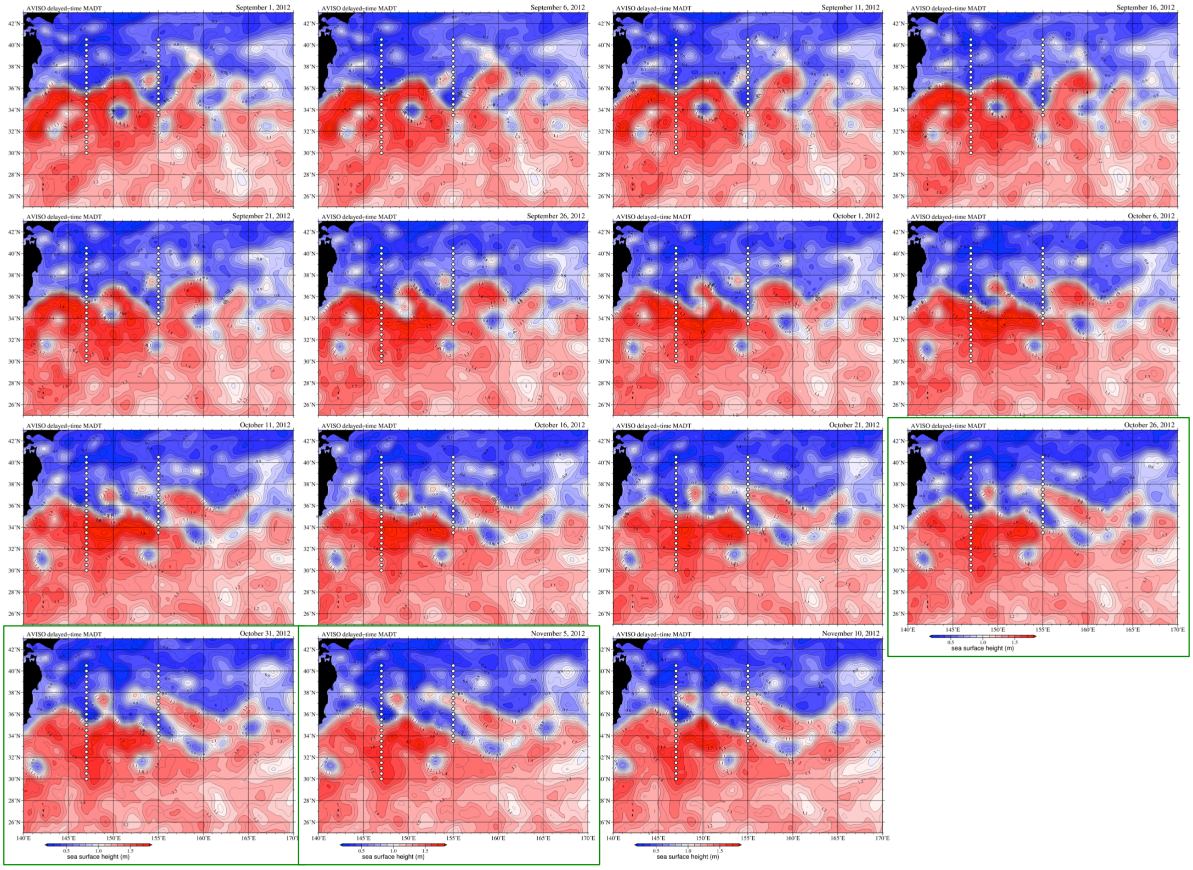

Figure S1. Temporal changes in SSH of the study area. SSH images are shown at 5-day intervals from 1 September to 10 November 2012. White circles are sampling locations for radiocaesium measurements during October and November 2012. Images during the sampling period are enclosed in green squares. The SSH data were produced by the Segment Sol Multimissions d'Altimétrie d'Orbotographie et de Localisation Précise/Data Unification and Altimeter Combination System and distributed by the Archiving, Validation and Interpretation of Satellites Oceanographic Data with support from the Centre National d'Etudes Spatiales (http://www.aviso.altimetry.fr/duacs/). The maps were made by using the General Mapping Tools version 4.5.11 (http://gmt.soest.hawaii.edu).

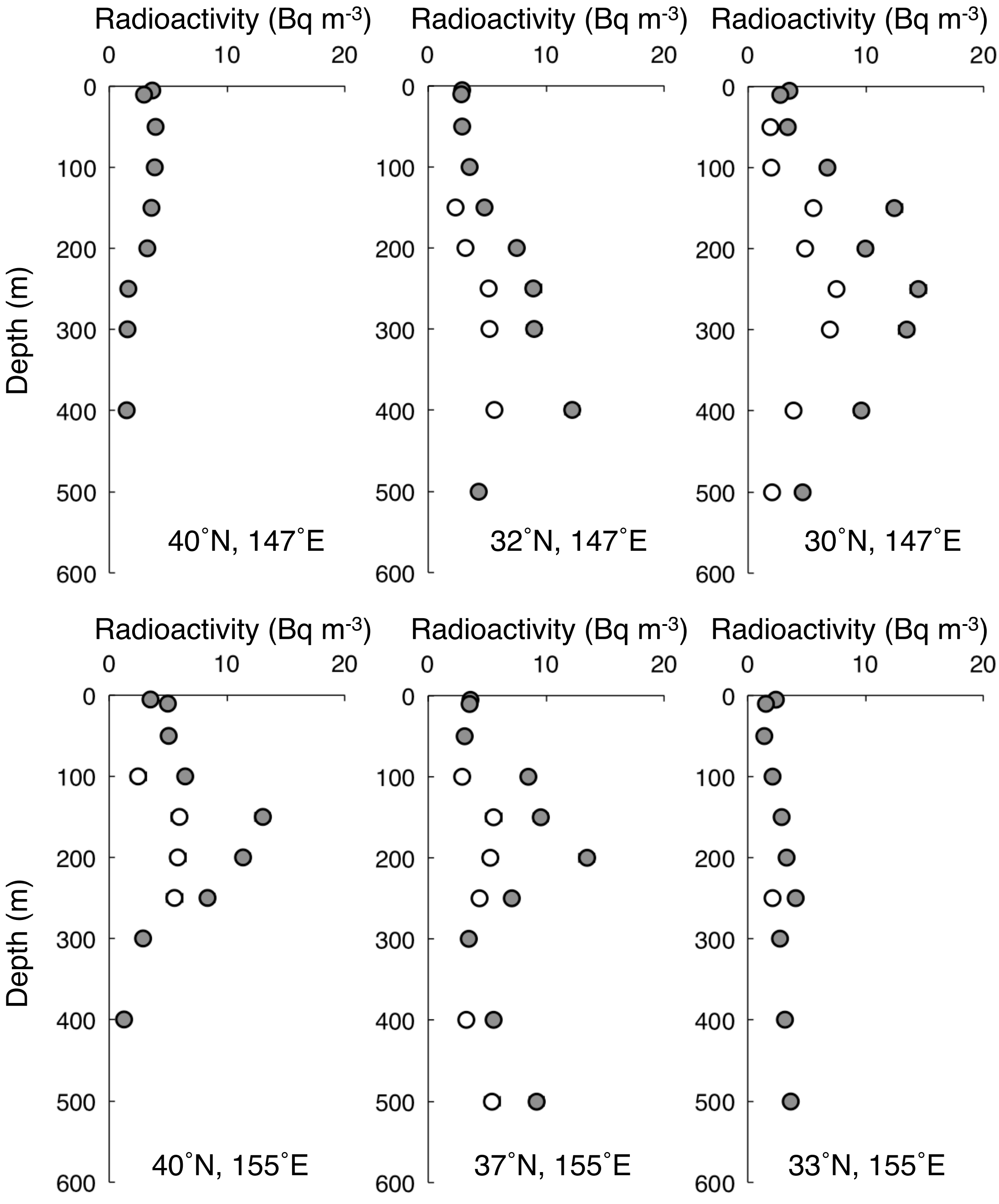

Figure S2. Vertical profile of ^{134}Cs and ^{137}Cs. Vertical profiles of ^{134}Cs and ^{137}Cs at representative stations, which located at north of KE, around KE and south of KE at 147˚E and 155˚E. White and grey circles represent concentration of ^{134}Cs and ^{137}Cs, respectively. Error bars indicate 1σ of counting error.

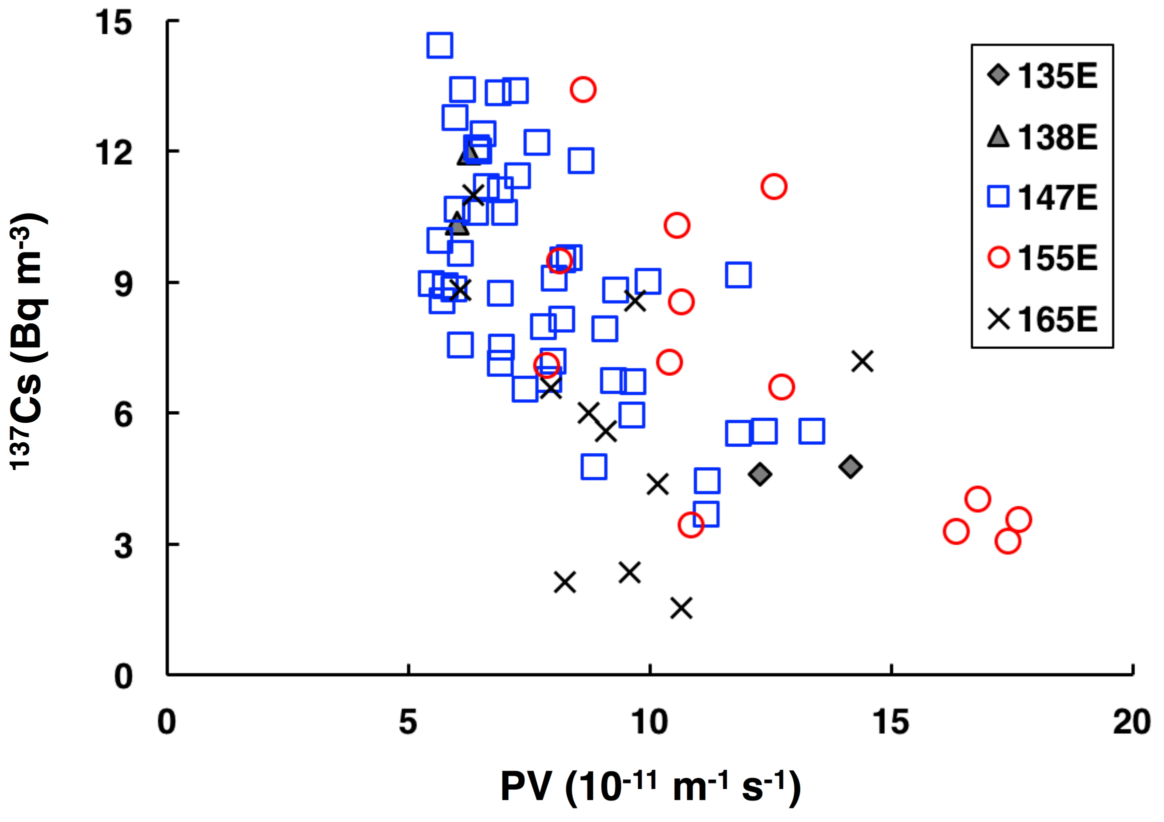

Figure S3. Relationship between ^{137}Cs and PV. Scatter plot of PV and ^{137}Cs concentration within the STMW (25.0-25.6 σ_θ). Blue squares and red circles indicate activities obtained along 147°E and 155°E, respectively, during October–November 2012. Grey diamonds and triangles indicate activities obtained along approximately 135°E from 12°N to 25°N in September 2012 and along 135°E from 27°N to 30°N in October 2012, respectively[24]. Crosses indicate activities obtained along 165°E from 8°N to 40°N in June 2012[30].
